# Exploring How Neighborhood Characteristics and Psychosocial Resilience Affect Cognition Among Older Black Adults

**DOI:** 10.1093/geroni/igab046.369

**Published:** 2021-12-17

**Authors:** Heather Farmer, Amy Thierry, Marina Armendariz, Sydney Kirven, Kyler Sherman-Wilkins

**Affiliations:** 1 University of Delaware, Newark, Delaware, United States; 2 Xavier University of Louisiana, Xavier University of Louisiana, Louisiana, United States; 3 The Pennsylvania State University, University Park, Pennsylvania, United States; 4 Xavier University of Louisiana, New Orleans, Louisiana, United States; 5 Missouri State University, Springfield, Missouri, United States

## Abstract

Black older adults are at greater risk for poor cognitive health than Whites, and adverse neighborhood conditions may contribute to this disparity. Moreover, limited research examines how resilience is implicated in the relationship between neighborhoods and cognition among Blacks. Using 2006-2016 waves of the Health and Retirement Study, we examine how perceived neighborhood characteristics (physical disorder and social cohesion) and psychosocial resilience (social support, mastery, and sense of purpose) contribute to cognitive functioning among 1,655 Black adults ages 65+. Results from multilevel linear regression models show that greater physical disorder was associated with worse cognitive functioning, and this was attenuated after adjustment for socioeconomic status. We found a positive association between purpose and mastery with cognitive functioning, even after accounting for socioeconomic, psychosocial, and health-related characteristics. Thus, high levels of purpose and mastery may be protective for cognitive functioning among Black older adults in spite of experiencing negative neighborhood contexts.

